# Functional Human and Murine Tissue‐Engineered Liver Is Generated from Adult Stem/Progenitor Cells

**DOI:** 10.5966/sctm.2016-0205

**Published:** 2016-08-30

**Authors:** Nirmala Mavila, Andrew Trecartin, Ryan Spurrier, Yi Xiao, Xiaogang Hou, David James, Xiaowei Fu, Brian Truong, Clara Wang, Gerald S. Lipshutz, Kasper S. Wang, Tracy C. Grikscheit

**Affiliations:** ^1^ Division of Gastroenterology, Department of Medicine, Cedars Sinai Medical Center, Los Angeles, California, USA; ^2^ Developmental Biology and Regenerative Medicine Program, Saban Research Institute, Division of Pediatric Surgery, Children's Hospital Los Angeles, Keck School of Medicine, University of Southern California, Los Angeles California, USA; ^3^ Department of Pathology and Laboratory Medicine, Children's Hospital Los Angeles, University of Southern California, Los Angeles, California, USA; ^4^ Department of Molecular and Medical Pharmacology and Department of Surgery, David Geffen School of Medicine at University of California, Los Angeles, Los Angeles, California, USA

**Keywords:** Tissue engineering, Liver regeneration, Organoid units, Liver transplantation, Liver failure

## Abstract

Liver disease affects large numbers of patients, yet there are limited treatments available to replace absent or ineffective cellular function of this crucial organ. Donor scarcity and the necessity for immunosuppression limit one effective therapy, orthotopic liver transplantation. But in some conditions such as inborn errors of metabolism or transient states of liver insufficiency, patients may be salvaged by providing partial quantities of functional liver tissue. After transplanting multicellular liver organoid units composed of a heterogeneous cellular population that includes adult stem and progenitor cells, both mouse and human tissue‐engineered liver (TELi) form in vivo. TELi contains normal liver components such as hepatocytes with albumin expression, CK19‐expressing bile ducts and vascular structures with α‐smooth muscle actin expression, desmin‐expressing stellate cells, and CD31‐expressing endothelial cells. At 4 weeks, TELi contains proliferating albumin‐expressing cells and identification of β2‐microglobulin‐expressing cells demonstrates that the majority of human TELi is composed of transplanted human cells. Human albumin is detected in the host mouse serum, indicating in vivo secretory function. Liquid chromatography/mass spectrometric analysis of mouse serum after debrisoquine administration is followed by a significant increase in the level of the human metabolite, 4‐OH‐debrisoquine, which supports the metabolic and xenobiotic capability of human TELi in vivo. Implanted TELi grew in a mouse model of inducible liver failure. Stem Cells Translational Medicine *2017;6:238–248*

**Significance Statement**

The worldwide burden of liver disease is approximately 30 million cases. In 2010, there were more than 1 million global deaths from one cause of liver disease alone, cirrhosis. The only effective therapy for end‐stage liver failure is liver transplantation, which is profoundly limited by scarce donor supply. In some conditions, such as inborn errors of metabolism or transient states of liver insufficiency, patients may be salvaged by providing partial quantities of functional liver tissue. In this study, it was hypothesized that a hardy multicellular cluster, or liver organoid unit, could be extracted from donor livers, which, after transplantation, would generate functional tissue‐engineered liver. This novel cellular therapy would have several proposed advantages: an accessible in vivo hepatic replacement that can be monitored, more efficient stem/progenitor cell production (perfusion is not required and cellular loss is minimized), and durable function. This approach could represent a personalized future therapy that would not require cellular reprogramming or immunosuppression to cure end‐stage liver diseases.

## Introduction

The worldwide burden of liver disease is approximately 30 million patients, affecting 1 in 10 people in the United States. In 2010, there were more than 1 million global deaths from one cause of liver disease alone, cirrhosis 1. The only effective therapy for end‐stage liver failure is liver transplantation, which is profoundly limited by scarce donor supply; only approximately 25,000 liver transplants per year occur worldwide. However, some forms of liver failure do not require replacement of the entire organ. In a number of congenital metabolic disorders, replacement of 5%–10% of the native liver mass may salvage the patient from the buildup of toxic metabolites 2 3 4 5 6. Therefore, an alternate approach to patients from end‐stage liver failure has been investigated, which we term “conventional liver cell transplantation” (CLCT). In CLCT, adult liver stem cells are transplanted, usually via the portal vein into the damaged liver. After CLCT, cells survive in general from 1–12 months, after which many patients still require liver transplantation 2. The donor livers required for these approaches remain scarce, and cells derived for CLCT are obtained through a perfusion protocol in which many cells are discarded. In CLCT, transplantation via the portal vein into the failing liver exposes the transplanted cells to an altered or possibly toxic milieu while also rendering the transplanted cells inaccessible for surveillance.

Additional future approaches include the possible application of human‐induced pluripotent stem cells (hiPSCs) but current progress with hiPSCs has been limited by the immaturity of some of the hepatocyte‐like cells generated from hiPSCs 7. In addition, unless the hiPSCs are derived from the liver failure patient's tissue, which is expensive and difficult to fully regulate, immunosuppression would still be required and off‐target effects including formation of other tissue types or malignancies is possible.

Based on our expertise in generating tissue‐engineered intestine (TEI) from adult stem cell populations 8 9 10 11 12 13 14 15, we have determined that profound cellular disruption, particularly of some epithelial/mesenchymal relationships is deleterious. In a variation of our process first developed to produce TEI, we hypothesized that we could extract a hardy multicellular cluster, or liver organoid unit (LOU), from donor livers, which, after transplantation, would generate functional tissue‐engineered liver (TELi), a novel cellular therapy, with several proposed advantages: an accessible in vivo hepatic replacement that can be monitored, more efficient stem/progenitor cell production (perfusion is not required and cellular loss is minimized), and durable function. In addition, if these cells are derived from autologous tissue, for example in children with short bowel syndrome in whom impending liver failure is predicted, stored cells could represent a future therapy that would not require cellular reprogramming or immunosuppression.

## Materials and Methods

All animal experiments were carried out after protocol approval by the Institutional Animal Care and Use Committee. Human liver samples were collected under an institutional review board‐approved protocol from the uninvolved margin of liver tumor resections or the explants obtained in the course of human liver transplantations for indications of liver failure.

### LOU Preparation

LOU were prepared from human liver or from 2‐week‐old Actin^GFP^ 16 mice and implanted in either nonobese diabetic (NOD)/severe combined immunodeficient (SCID) or Rag2−/−Il2rg−/−Arg^flox/flox^ UBC‐Cre/ERT2 17 mice in a variation of the protocol we have developed for regions of the intestine 18. Briefly, under sterile conditions, resected liver was cut into approximately 2‐ to 3‐mm or smaller pieces and washed in ice cold Hank's balanced salt solution, sedimented between washes with centrifugation (150*g*), and then digested in either 800 or 11,000 U/ml of collagenase type I (Worthington Biochemical Corporation, Lakewood, NJ, http://www.worthington-biochem.com) and 0.12 mg/ml of dispase (Thermo Fisher Scientific, Waltham, MA, http://www/thermofisher.com). LOU were pelleted and washed twice in Dulbecco's modified Eagle's medium (DMEM), the first solution containing 10% fetal bovine serum. After the final wash in DMEM, organoid units (OU) were pelleted by centrifugation at 150*g* for 5 minutes at 4°C, and the resultant pellet containing LOU was either vitrified or dispersed on a biodegradable scaffold for immediate implantation. Vitrification was performed as described by Spurrier et al. 19.

### Scaffold Preparation

Scaffolds were prepared as previously published 18. Briefly, nonwoven polyglycolic acid scaffolds are rolled over a 1.5‐mm outer diameter glass capillary tube from a flat sheet (2‐mm sheet thickness, 60 mg cm^−3^ bulk density; porosity >95%; Concordia Fibers, Coventry, RI. http://www.concordiafibers.com). The entire cylinder is sealed with 5% poly‐L‐lactic acid (Durect Corporation, Cupertino, CA, http://www.durect.com) in chloroform (Sigma‐Aldrich, St. Louis, MO, https://www.sigmaaldrich.com). After sterilization with 80% ethanol, the polymers were coated with 0.4 mg/ml type 1 collagen solution (Sigma‐Aldrich) for 20 minutes at 4°C, rinsed with phosphate buffered saline (Thermo Fisher), and stored dry at room temperature in a desiccator (Durapore 0.45 μm; Millipore, Billerica, MA, http://www.emdmillipore.com) to avoid premature hydrolysis, degradation or contamination. Fresh cut constructs (3 mm) were used for implantations 18.

### Implantation

Under sterile conditions, approximately 1 × 10^5^ LOU/scaffold were loaded on to biodegradable scaffolds and directly implanted under the abdominal skin of 3‐ to 6‐month‐old mice. NOD/SCID γ host mice (catalog no. 005557; The Jackson Laboratory, Sacramento, CA, https://www.jax.org) received full‐body irradiation (350 cGy) before implantation. Scaffold loading was consistent with 60 μl of LOU‐containing medium transferred by sterile micropipette onto each scaffold. This volume contains approximately 1× 10^5^ OU, as we have previously determined that this is a range that grows well. Because OU are multicellular, the actual cell number will vary. Under anesthesia and sterile conditions, the abdominal skin was opened approximately 1 cm without violating the rectus muscle. Scaffolds containing LOU were implanted under the skin and the incision closed with 4‐0 Vicryl sutures (Ethicon). Mice were given free access to (Irradiated Laboratory Diet 5061, PMI Nutrition, Brentwood, MO) and water with Septra 200 mg / 40 mg per 5 ml (Hi‐Tech Pharmacal) at 1:100 dilution. After euthanasia, blood draws were performed for the serum albumin assay and mass spectrometric analysis of drug metabolite at 4‐week time points at the time of TELi harvest via cardiac puncture.

### Vitrification

Vitrification was performed as described in 19. Briefly, murine or human LOU were suspended in stepwise increasing concentrations of DMSO (0.5 M, 1 M, and 2 M), and supercooled in an ethanol bath to −13°C. Subsequently, they were nucleated for vitrification. Latent heat was released for 10 minutes at −13°C and samples were cooled to −80°C at a controlled rate of −1°C/min, then plunged into liquid nitrogen for long‐term storage. Thawing was rapid (150°C–200°C/minute) via agitation in a 37°C water bath. OU were then transferred to a 15‐ml tube in an ice bath, and 1 ml of 0.75 M sucrose in Hanks’ balanced salt solution (HBSS) was added. After 5 minutes, the tube was centrifuged at 100*g* for 2 minutes at 4°C. The sample was returned to the ice bath, supernatant was discarded, and an additional 1 ml of 0.75 M sucrose in HBSS was added. After 5 minutes, 1 ml of plain HBSS was added to the solution, decreasing the sucrose concentration by half. After 5 minutes, another 2 ml of HBSS was added, followed by 4 ml after 5 additional minutes. The solution was again centrifuged at 150*g* for 10 minutes at 4°C, and the supernatant was discarded.

### LOU Cellular Identification

LOU were mounted in agarose as in 19, with immunohistochemistry or immunofluorescence as described later.

### TELi Cellular Identification

TELi was harvested at 4 weeks except for the arginase deficiency experiments, when the TELi was harvested 2 weeks after all animals usually succumb from liver failure in this model. Explanted TELi constructs were fixed overnight in formalin (Thermo Fisher) and processed for paraffin embedding. Explanted TELi were also frozen for RNA analysis and stored at −80°C until analysis. Five‐micrometer‐thick cross‐sections were stained with hematoxylin and eosin (H&E) according to standard procedures. Immunofluorescence staining on tissue sections was performed as described 20. Five‐micrometer‐thick sections were used for immunofluorescence staining. For three‐dimensional histology staining, 50‐μm‐thick sections were used. Briefly, after antigen retrieval, tissue sections were incubated with primary antibodies that were diluted with 5% normal goat serum prepared in Tris‐buffered saline Tween 0.5% overnight at 4°C. Different liver cell types were identified based on its specific markers such as albumin, hepatocyte nuclear factor‐α (HNF4α), CK8 (hepatocytes), CK19 (cholangiocytes), desmin (stellate cells), CD31 (vascular endothelial cells), and α−smooth muscle actin (SMA) (portal fibroblast cells). Proliferating cells were determined by staining for proliferating cell nuclear antigen (PCNA). Complete list of antibodies and dilutions used in this study are noted in supplemental online Table 1. Fluorescent signals were detected by staining with Cy3, Cy5, or fluorescein isothiocyanate‐conjugated secondary antibodies (Jackson Immuno Research Labs, West Grove, PA, https://www.jacksonimmuno.com). Tissue sections were mounted with Vector shield containing 4′,6‐diamidino‐2‐phenylindole (DAPI; Vector Laboratories, Burlingame, CA, https://vectorlabs.com). Images were acquired using an upright fluorescent microscope (Leica DM5500). Staining was performed at least three or more times on independent tissue sections.

### Reverse Transcription‐Polymerase Chain Reaction

RNA was isolated from frozen TELi with the Qiagen RNA purification kit. cDNA was prepared from 1000 ng of RNA using Bio‐Rad cDNA synthesis kit (Bio‐Rad, Hercules, CA, http://www.bio-rad.com). Reverse transcription‐polymerase chain reaction (RT‐PCR) was performed to detect the gene expression of albumin, CK18 and HNF4α. Primer sequences are given in supplemental online Table 1.

### Serum Human Albumin Enzyme‐Linked Immunosorbent Assay

Blood from TELi implanted and control mice were collected by cardiac puncture on time points as describe previously, spun down in a heparin gel containing tube for plasma separation. Human albumin in the plasma was quantified by human albumin enzyme‐linked immunosorbent assay kit (Bethyl Laboratories, Montgomery, Texas, https://www.bethyl.com) according to the manufacturer's instructions.

### Human Drug Metabolism

The xenobiotic metabolizing capacity was evaluated with in vivo administration of debrisoquine as described previously 21, 22. Debrisoquine is primarily metabolized by human enzyme CYP2D6 into 4‐OH‐debrisoquine, and was administered via oral gavage before human TELi (hTELi) harvest. Blood was collected in heparin tubes (BD Biosciences, San Jose, CA, http://www.bdbiosciences.com) at 1 and 4 hours after drug administration. Plasma was separated by centrifugation. Internal standards (niflumic acid, 1 μM) in methanol solution (100 μl) was added to 10 μl of serum and the supernatant after centrifugation (16,000*g*, 4°C, 5 minutes) was subjected to liquid chromatography/mass spectrometric analysis. Chromatographic separation was achieved with a Waters Acquity BEH C18 column (2.5 μm, 3 × 150 mm; Waters Corporation, Milford, MA, http://www.waters.com) with a Thermo Ultimate 3000 UHPLC system (Thermo Fisher). Column temperature was maintained at 50°C and the flow rate was 0.9 ml/min. Mobile phase A consisted 0.1% formic acid in water, and mobile phase B consisted 100% acetonitrile. The gradient started with an initial condition of 3% mobile phase B for 1.5 minutes, followed by a linear gradient to 97% mobile phase B over 3 minutes. This condition was maintained for 2 minutes, returned to the initial condition over 0.1 minute, and maintained until the end of a 9‐minute run. The liquid chromatography was connected to a Thermo Quantiva mass spectrometer (Thermo Fisher) operated in positive electrospray ionization mode. Quantitation was based on multiple reaction monitoring mode with the following parameters: spray voltage, 2,100 V; sheath gas pressure, 55 arb; auxiliary gas pressure, 13 arb; sweep gas pressure, 3 arb; ion transfer tube temperature, 375°C; a vaporizer temperature, 480°C. Debrisoquine was quantified with a mass transition of 176.3 to >134.1 (m/z) and 4‐OH‐debrisoquine was quantified with a mass transition of 192.3 to >132.1 (m/z). Other production ions were also monitored to confirm the identities of the metabolite and parent drug. Percentage metabolite conversion was then calculated and compared.

### Arginase Deficiency Model

Six Fah−/− Fah−/−Rag2−/−Il2rg−/−Arg^flox/flox^ UBC‐Cre/ERT2 17 female mice were implanted with 6 implants each derived from Actin^GFP^ mice 16. Six weeks later, 100 μl tamoxifen (20 mg/ml) (Sigma‐Aldrich), prepared in corn oil, was administered by oral gavage for 10 days. Blood was drawn for serum amino acid measurement at euthanasia. Native livers of the Fah−/− Fah−/−Rag2−/−Il2rg−/−Arg^flox/flox^ UBC‐Cre/ERT2 mice rescued with TELi were stained for arginase to confirm that arginase deficiency had been induced as in 17 and enzymatic quantification of the urea/micrograms protein of the native livers of the Fah−/− Fah−/−Rag2−/−Il2rg−/−Arg^flox/flox^ UBC‐Cre/ERT2 mice was performed as in 17 to confirm induction of the normally lethal inducible arginase 1 deficiency state.

### Metabolite and Ammonia Analysis

Plasma amino acid analysis was performed on a Biochrom 30 HPLC amino acid analyzer (Biochrom, Cambridge, United Kingdom, http://www.biochrom.co.uk) as previously described 23. Ammonia determination was performed from plasma samples per manufacturer's instructions (Abcam, Cambridge, MA, http://www.abcam.com). Briefly, after equilibrating all reagents at room temperature, standards were prepared. Samples were set up using 5 μl of plasma added to 45 μl of assay buffer in duplicate in a 96‐well microplate. The reaction mix was prepared as a master mix with each reaction consisting of 42 μl ammonia assay buffer, 2 μl OxiRed Probe, 2 μl Enzyme Mix, 2 μl developer, and 2 μl converting enzyme. Fifty microliters of reaction mix was added to samples and standard wells, mixed, and incubated for 1 hour at 37°C in a dark container. Optical density was measured at 570 nm using a microplate reader (iMark 16704; Bio‐Rad). From the standards, a line was generated and sample results in mM were calculated.

### Statistical Analysis

An unpaired Student's *t* test was performed to determine the statistical significance. A *p* value of >.05 was considered as significant. A Grubbs’ test with α = 0.05 removed outliers before the *t* test was performed.

## Results

### Murine LOU Generated TELi, Which Consists of Key Cell Types That Are Necessary for Hepatic Function, but the Cellular Organization Does Not Directly Recapitulate Native Liver Structure

**Figure 1:**
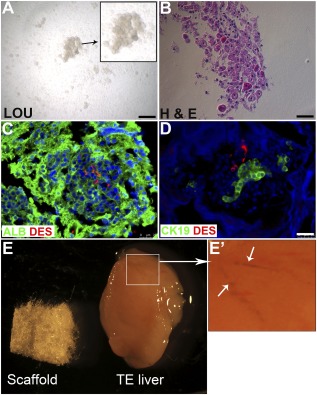
LOU generate TE liver (TELi). **(A):** Phase contrast image of LOU. **(B):** H&E staining. **(C–D):** Immunofluorescence staining for albumin (green, hepatocytes); desmin (red, stellate cells); and CK19 (green, cholangiocytes) in LOU. Nuclei stained with 4′,6‐diamidino‐2‐phenylindole (blue). Scale bar = 25 μm. **(E):** Biodegradable scaffold and TELi harvested at 4 weeks. **(E′):** Presence of branched capillaries on the surface of TELi. Images are representative of multiple experiments. Abbreviations: ALB, albumin; DES, desmin; H&E, hematoxylin and eosin; LOU, liver organoid units; TE, tissue engineered.

**Figure 2:**
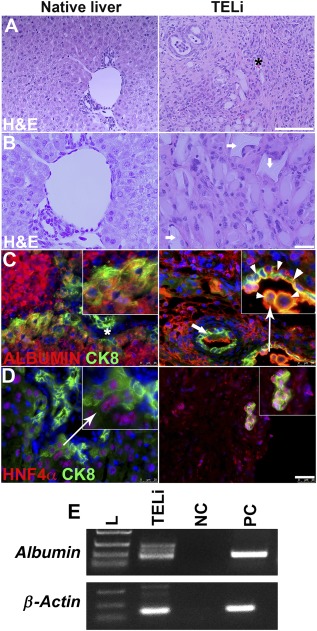
Immunohistologic analysis of TELi. **(A–B):** H&E staining of native liver and TELi. An asterisk is placed near a blood vessel containing erythrocytes in TELi. White arrows indicate incompletely degraded scaffold material. **(C):** Immunofluorescence staining for albumin (red) and CK8 (cholangiocyte and periductular hepatocyte marker, green) in native liver and TELi. Arrowhead shows double positive cells. **(D):** Immunofluorescence staining for HNF4α (hepatocyte specific transcription factor, red) and CK8 (green). Nuclei stained with 4′,6‐diamidino‐2‐phenylindole (blue). **(E):** Reverse transcription‐polymerase chain reaction for albumin expression. cDNA prepared from whole liver RNA. Scale bars = 50 μm **(A)**, 25 μm **(B–D)**. Data represent more than three independent experiments. Abbreviations: H&E, hematoxylin and eosin; L, ladder; NC, negative control (water); PC, positive control; TELi, tissue‐engineered liver.

The multicellular LOU (Fig. 1A, 1B) contains hepatocytes and various nonparenchymal cell types (Fig. 1C, 1D) that are necessary for liver function. Implantation of LOU results in the generation of TELi that expresses albumin robustly, comparable to the native liver (Fig. 2C). The presence of hepatocytes was further confirmed by the expression of hepatocyte specific transcription factor, HNF4α in TELi (Fig. 2D). Costaining further demonstrated that TELi contains cells that coexpress albumin or HNF4α with CK8 (Fig. 2C, 2D). CK8 is a type of cytokeratin that is known to express in biliary cells and periportal hepatocytes. Vitrified human LOU also generated TELi containing hepatocytes on H&E staining and host serum analysis confirmed their synthetic function.

### TELi Contains Key Liver Cell Types but Has a Different Organization

**Figure 3:**
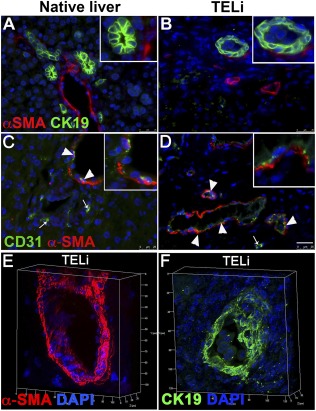
TELi contains patent vascular and biliary structures. **(A, B):** Immunofluorescence staining for α‐SMA (portal fibroblasts, red) and CK19 (cholangiocytes, green). **(C, D):** Immunofluorescence staining for CD31 (vascular endothelial cells, green) and α‐SMA (portal fibroblasts; red). Arrowhead shows double positive cells. Arrows point to single positive cells. **(E, F):** Immunofluorescence staining for α‐SMA and CK19 in a three‐dimensional z‐stack analysis of TELi demonstrates patent vascular and biliary ductal structures. Nuclei stained with DAPI (blue). Scale bar = 25 μm **(A–D)**. Images represent three to four independent experiments. Abbreviations: α‐SMA, α‐smooth muscle actin; DAPI, 4′,6‐diamidino‐2‐phenylindole; TELi, tissue‐engineered liver.

On H&E (Fig. 2A, 2B) TELi contains cylindrical structures consistent with blood vessels and biliary tubules that appear to have a patent lumen on confocal imaging, (Fig. 3E, 3F). But the overall structure of TELi does not recapitulate the normal portal triads as seen in native liver: Although smaller blood vessels are readily identified, no large outflows for either the biliary or vascular system were identified in the collective experiments, and cholestasis, or pigmented hepatocytes that contain undrained bile, was at times recognized (Fig. 2A, 2B; supplemental online Fig. 1A, 1B, arrows). At 4 weeks, a small amount of degrading polymer was also observed (Fig. 2B, arrows).

### In TELi, Hepatocytes Are Similar to Those in Native Liver

Hepatocytes are the major epithelial cell type that performs the metabolic functions of the liver. TELi expresses albumin robustly, comparable to the native liver (Fig. 2C). The presence of hepatocytes was further confirmed by the expression of hepatocyte specific transcription factor, HNF4α in TELi (Fig. 2D). Costaining further demonstrated that TELi contains cells that coexpress albumin or HNF4α with CK8 (Fig. 2C, 2D). CK8 is a type of cytokeratin that is known to express in biliary cells and periportal hepatocytes. To exclude the possibility of host liver‐derived albumin in TELi, RT‐PCR was performed on RNA isolated from the explanted TELi albumin RNA transcripts were identified in TELi (Fig. 2E).

### TELi Contains Smaller Generations of Patent Bile Ducts and Blood Vessels as Well as Stellate Cells

Branched capillaries are visible on the surface of TELi at the time of harvest (Fig. 1E, 1F). Immunofluorescence staining for CK19, a marker for mature cholangiocytes, indicates the presence of bile ducts in TELi (Fig. 3A). Multilayer tissue analysis by z‐stack imaging further demonstrated that vessels and bile ducts possess clear lumens (Fig. 3E, 3F). The presence of CD31‐expressing endothelial cells and α‐SMA‐expressing portal fibroblast cells demonstrated the presence of vascular structures as seen in native liver (Fig. 3C, 3D, arrowheads). Immunofluorescence staining was performed for the stellate cell marker, Desmin and identified stellate cells that were similar to those marked in native liver ( supplemental online Fig. 2).

### Hepatocytes Proliferate in TELi

**Figure 4:**
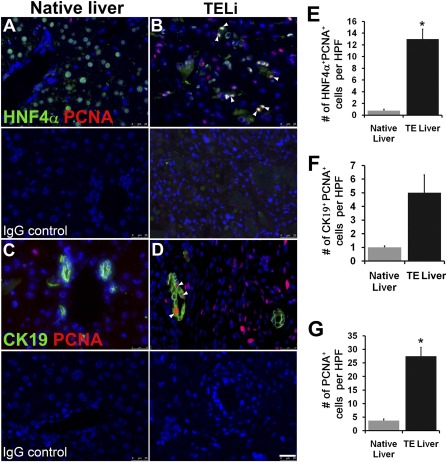
TELi contains proliferative cells. **(A, B):** Immunofluorescence costaining for PCNA and HNF4α in native liver and TELi. **(C, D):** Immunofluorescence staining for PCNA and CK19. Arrow shows copositive cells. **(E–G):** Quantification of double‐positive cells **(E, F)** and PCNA single‐positive cells **(G)** per HPF. ∗, *p* < .05, *n* = 4. Scale bar = 25 μm. Abbreviations: HNF‐α, hepatocyte nuclear factor‐α; HPF, high‐power field; PCNA, proliferating cell nuclear antigen; TE, tissue engineered; TELi, tissue‐engineered liver.

Staining for PCNA demonstrated a significant increase of approximately 7‐fold in total proliferating cells (Fig. 4A–4G, arrowheads) and a significant increase of approximately 12‐fold in PCNA^positive^HNF4α^positive^ cells (Fig. 4A, 4B, 4E). The majority of the hepatocytes in TELi are small‐nucleated cells compared with native hepatocytes, which have a comparatively larger nucleus. PCNA costaining with CK19 demonstrated a nonsignificant increase in proliferating cholangiocytes in TELi compared with the native liver (Fig. 4C, 4D, 4F). Immunostaining of TELi demonstrated the presence of SOX9 only within the bile ducts as seen in native liver and was absent in the parenchyma of TELi ( supplemental online Fig. 3).

### TELi Develops From Implanted LOU

**Figure 5:**
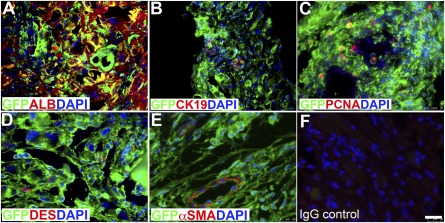
Immunofluorescence costaining for GFP (donor cells) and cell‐specific markers. **(A):** Albumin for hepatocytes; **(B):** CK19 for cholangiocytes **(C):** PCNA for proliferating cells; **(D):** desmin for hepatic stellate cells; **(E):** α‐SMA for portal fibroblast cells. **(F):** IgG was the negative control. Nuclei stained with DAPI (blue). Scale bar = 25 μm. Images represent three to four independent experiments. Abbreviations: ALB, albumin; α‐SMA, α‐smooth muscle actin; DAPI, 4′,6‐diamidino‐2‐phenylindole; DES, desmin; GFP, green fluorescent protein; PCNA, proliferating cell nuclear antigen.

To determine the origin of various cell types in TELi, we performed costaining for various cell‐specific markers and green fluorescent protein (GFP), which marked our donor cells in LOU when derived from Actin^GFP^ mice. Epithelial cells such as hepatocytes (albumin‐positive), cholangiocytes (CK19‐positive), and mesenchymal cells such as stellate cells (desmin‐positive) and portal fibroblast cells (α‐SMA‐positive) were also positive for GFP, indicating their origin from implanted LOU (Fig. 5A–5E). Further costaining with PCNA and GFP demonstrates that implanted cells are proliferating in TELi at 4 weeks (Fig. 5C). Few alpha‐fetoprotein (AFP)‐positive cells were identified by immunofluorescent staining of TELi, with still fewer in native liver, consistent with repair/regeneration in TELi as compared with native tissue homeostasis ( supplemental online Fig. 4) When comparing immunofluorescent detection of stem cell marker CD133 and liver stem/progenitor cell marker epithelial cell adhesion molecule (EpCAM), more positive cells were noted in TELi than native liver ( supplemental online Fig. 5).

### Human Liver Organoid Units Develop Into TELi in a Murine Model

**Figure 6:**
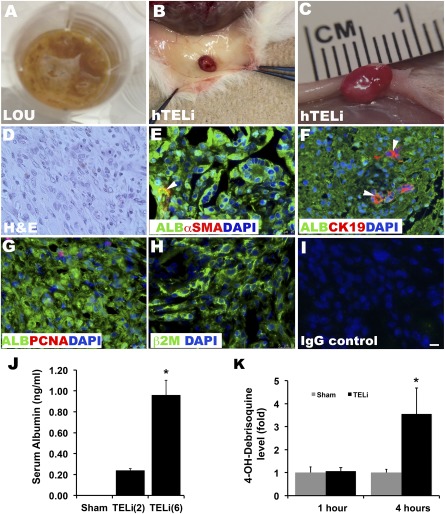
Human LOU generate human TELi. **(A):** Human LOU prepared from fresh liver biopsy samples. **(B–C):** hTELi in murine host at 4 weeks. **(D):** H&E of cellular hTELi at 4 weeks. **(E):** Immunofluorescence costaining for ALB and α‐SMA. **(F):** Costaining for ALB and CK19. **(G):** Costaining for ALB and PCNA. **(H):** Human antigen β2M. **(I):** IgG staining control. Nuclei stained with DAPI (blue). Scale bar = 25 μm. Images represent three to four independent experiments. **(J):** Quantification of human ALB in murine plasma by enzyme‐linked immunosorbent assay. Number in parentheses is number of implanted TELi in each mouse. Sham, *n* = 4; TELi(2), *n* = 4, TELi(6), *n* = 13. ∗, *p* < .05 compared with TELi(2). **(K):** Liquid chromatography‐mass spectrometric quantification of human specific metabolite, 4‐OH‐debrisoquine derived from debrisoquine in vivo in mice with hTELi. One hour and 4 hours on the x‐axis indicate time after debrisoquine administration in mice. Sham, *n* = 4‐6; TELi, *n* = 7–11. ∗, *p* < .05 compared with 4‐hour sham. One‐hour TELi to 1‐hour sham was not significant (*p* = .08). Abbreviations: ALB, albumin; α‐SMA, α‐smooth muscle actin; β2M, β2‐microglobulin; DAPI, 4′,6‐diamidino‐2‐phenylindole; H&E; hematoxylin and eosin; hTELi, human tissue‐engineered liver; LOU, liver organoid units; PCNA, proliferating cell nuclear antigen; TELi, tissue‐engineered liver.

In an effort to generate hTELi, human LOU were prepared from human liver tissue that would otherwise have been discarded in the operating room, under an institutionally approved protocol. After 4 weeks, donor LOU developed into full thickness hTELi, more than twice the size of the original implanted scaffolds (Fig. 6B, 6C). Hematoxylin and eosin staining demonstrated cell types comparable with the normal liver, however, as noted previously, with less organization (Fig. 6D). Immunofluorescence staining demonstrated presence of albumin‐expressing hepatocytes, CK19‐expressing biliary epithelium (Fig. 6F), and α‐SMA‐positive vascular structures (Fig. 6E). PCNA staining demonstrated that hepatocytes are proliferating in hTELi (Fig. 6D). To confirm the human origin of cells in the hTELi, staining for human‐specific protein, β2 microglobulin was performed. There are abundant β2‐microglobulin‐positive cells were present in the hTELi, confirming the contribution of implanted human cells in the formation of hTELi (Fig. 6H).

### Human Tissue‐Engineered Liver Is Functional In Vivo

To determine the secretory and metabolic function of hTELi, plasma was collected from hTELi‐implanted host mice at 4 weeks, before tissue harvest. Sham‐operated mice served as controls. There was no human albumin detected in control mice who hosted no implants. A significant fivefold increase in the serum human albumin level was observed in mice that had six hTELi implants (*n* = 6) compared with the mice (*n* = 4) that had two hTELi implants (Fig. 6J). To determine the drug metabolizing or xenobiotic capacity of hTELi, debrisoquine, a drug that is differentially metabolized by human hepatocytes, was administered to mice that had been implanted with LOU 4 weeks prior 24. Plasma was collected at two different time points to determine the relative level of the debrisoquine metabolite, 4‐OH‐debrisoquine. Plasma was processed and subjected to liquid chromatography/mass spectrometric analysis as described previously 24. One hour after drug administration, there was no significant change in serum 4‐OH‐ debrisoquine levels. However after 4 hours, a significant approximate 3.5‐fold increase in metabolite was detected (Fig. 6K; supplemental online Fig. 6).

### TELi Contributed to Some Hepatic Functions in Mice With a Lethal Form of Metabolic Disorder and TELi Contains Key Cell Types at 13 Weeks

**Figure 7:**
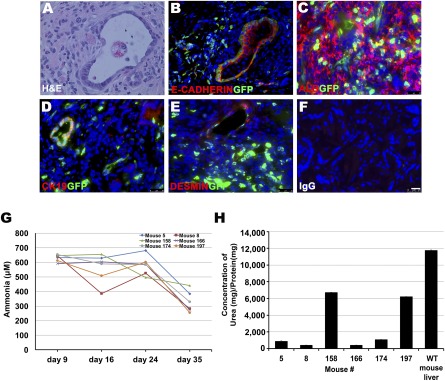
TELi grows in an arginase deficiency model. **(A):** H&E staining of TELi harvested from Rag2−/−Il2rg−/−Arg^flox/flox^ UBC‐Cre/ERT2 hosts. **(B):** Immunofluorescence staining demonstrates E‐cadherin‐positive and GFP‐positive cells in TELi harvested from arginase‐1‐deficient mice after 13 weeks. **(C):** ALB and GFP coimmunostaining of TELi. **(D):** CK19 and GFP coimmunostaining of TELi. **(E):** Presence of desmin‐expressing stellate cells **(F):** IgG control. **(G):** Serum ammonia levels were obtained at 9, 16, 24, and 35 days after tamoxifen induction. Historically, 100% of females die with hyperammonemia by day 23 after tamoxifen administration. Ammonia levels were decreased from baseline at the time of harvest on day 35. **(H):** Host native livers had variable but lower concentrations of urea. Scale bar = 25 μm **(A–F)**. Abbreviations: ALB, albumin; GFP, green fluorescent protein; H&E, hematoxylin and eosin; TELi, tissue‐engineered liver; WT, wild‐type.

TELi were implanted subcutaneously in transgenic mice that have an inducible lethal form of Arginase deficiency. Arginase‐1 (ARG‐1) deficiency is an autosomal recessive disorder that affects the urea cycle, leading to impaired ureagenesis 25. Mice that received TELi implants 6 weeks before induction of arginase deficiency survived a significantly longer period than reported historical controls (Fig. 7). Histologic analysis demonstrated that at 13 weeks, as at the 4‐week time point described previously, TELi contains hepatocytes and bile duct structures (Fig. 7B). Measurement of the concentration of urea/protein in the native livers of the host mice that were salvaged with the implantation of six TELi constructs each was variable although reduced from that of wildtype livers in every case. However, this variability requires consideration of contributions of clonal populations of hepatocytes that escaped induction of ARG1 deficiency in the mouse native liver in addition to consideration of functions of the implanted TELi. Reduction was lowest in four mice (numbers 5, 8, 166, and 174) and more modest in two mice (158 and 197) (Fig. 7D). The following ratios of urea to protein concentration and the standard deviation for each mouse were obtained: mouse number 5 was 908.5 ± 13.7; number 8 was 410.3 ± 6.4; mouse 158 was 6470.9 ± 3.6; mouse number 166 had 411.3 ± 8.6; mouse 158 was 1091.1 ± 5.7; mouse 174 was 6230.8 ± 7.1; compared with the wild type control of 11791.6 ± 2.9 (μg urea per mg protein). In all animals, the serum ammonias initially climbed and then, at the conclusion of the experiment at 13 weeks, had returned to lower values, consistent with TELi growth, but likely with concurrent clonal expansion of some hepatocyte populations in the native liver (Fig. 7C). This was confirmed with immunohistochemistry for arginase, which did indicate some expression in the native livers as well as in TELi (data not shown). Serum amino acids were measured at the conclusion of the experiment ( supplemental online Table 2). We also detected the Ki67 protein by immunofluorescence at the conclusion of the experiment to rule out uncontrolled proliferation. We saw sparse Ki67 staining at 13 weeks and no gross evidence of malignancy on histologic sectioning ( supplemental online Fig. 7).

## Discussion

### LOU Implantation on a Biodegradable Scaffold Generates TELi

The multicellular LOU contains multiple liver cell types including hepatocytes, biliary ductal cells, and stellate cells (Fig. 1C, 1D). Four weeks after implantation, LOU developed into TELi, whereas the biodegradable scaffold hydrolyzed and was cleared, although polymer fragments persisted at 4 weeks, but were less noticeable at 13 weeks in the rescue experiments. The TELi more than doubled in size from the implanted construct, with visible ingrowth of a supporting vasculature (Fig. 1E, 1F). We elected for a subcutaneous implantation site to ensure that our harvested constructs had no possibility of contamination with extrinsic native liver tissue. In addition, we hypothesized that implantation outside of the liver has some advantages: improved access for surveillance and exclusion from a potentially toxic milieu in the case of liver failure, the intended clinical scenario for future translation.

### Although TELi Contains Key Liver Cell Types, the Structure of the Native Liver Is Not Exactly Recapitulated

TELi contains nonsinusoidal blood vessels and biliary tubules (Fig. 2A, 2B) confirmed with confocal imaging, (Fig. 3E, 3F), with erythrocytes seen within lumen in some sections suggesting potential blood flow through vessels. The small bile ducts and vessels were demonstrably patent on confocal imaging (Fig. 3E, 3F). The small‐medium size of the developing blood vessels is unlikely to be a problem for the vascular system, as angiogenesis and vasculogenesis via small caliber networks is known to support large tissue volumes, but bile drainage in native liver converges onto a central common bile duct for drainage into the duodenum, and obstruction of this flow is known to be pathologic. Extensive cholestasis or trapping of pigmented bile acids within the hepatocytes was not observed, although it was identified in some clusters of hepatocytes within TELi ( supplemental online Fig. 1A, 1B). This may be due to lower activity of the hepatocytes within TELi actively extracting bile acids from the serum, our first hypothesis, as cholestasis was not markedly increased in the arginase deficiency model where the native liver of the animals implanted with TELi for 13 weeks demonstrated evidence of altered hepatic function. In future work, the effect of the disorganized structure on longer‐term function and biliary outflow will need to be investigated. This may require alternative configurations for implantation, including shortening the time for scaffold degradation, as at 4 weeks, a small amount of degrading polymer was also observed (Fig. 2B). In addition, in the case of tissue‐engineered intestine, longer periods of implantation have resulted in improved organ architecture; although we did not see a marked improvement in the cellular organization by 13 weeks in the rescue experiments, the timeline may be longer than we studied. In addition, there are current surgical drainage techniques for biliary obstruction, which could be employed if this is the limitation for human therapy.

### Although the Overall Structure of TELi Was Less Organized Than Native Liver, Key Cell Types Within TELi Were Identified

The hepatocytes within TELi expressed albumin and HNF4α, which costained with CK8 similarly to the native liver (Fig. 2C, 2D), and RT‐PCR identified albumin RNA transcripts in TELi (Fig. 2E). Liver stellate cells or Ito cells line the sinusoidal space and store vitamin A. Hepatic stellate cells play an important role in tissue regeneration by releasing growth factors 26. To confirm the presence of stellate cells in TELi, immunofluorescence staining was performed for the stellate cell marker, desmin. Stellate cells were noted in a location comparable with those identified in native liver ( supplemental online Fig. 2). When marked with GFP, the donor cells from LOU could be identified in the resulting TELi, including in hepatocytes, cholangiocytes, stellate cells, and fibroblasts cells adjacent to blood vessels (Fig. 5). Proliferation was assessed by staining for PCNA; PCNA and GFP costaining identified that GFP‐positive donor cells were proliferating in TELi at 4 weeks (Fig. 5), and there was a significant increase in total and PCNA^positive^HNF4α^positive^ proliferating cells (Fig. 4A–4E), although the cholangiocytes in TELi were not proliferating significantly compared with those in native liver (Fig. 4C, 4D). AFP, CD133, and EpCAM staining was also identified in discrete cells ( supplemental online Figs. 4, 5). SOX9 is a transcription factor expressed in the biliary epithelium. It is also expressed in a variety of progenitor cells in other organs. In the liver, SOX9 regulates maturation and maintenance of bile ducts. Liver‐specific deletion of SOX9 during murine liver development has been known to delay bile duct morphogenesis 27, 28. SOX9 was noted largely within biliary epithelium in TELi as in native liver ( supplemental online Fig. 3).

### Human Liver Organoid Units Develop Into hTELi in a Murine Model, Which Demonstrates Human‐Specific Function

hTELi was generated from both fresh and vitrified LOU, with similar key cell types as noted previously, including hepatocytes, biliary epithelium, and vascular structures as noted in murine TELi (Fig. 6). PCNA indicated proliferation of the hepatocytes within hTELi (Fig. 6G) and the human origin of the donor cells were confirmed by staining for the human specific protein, β2‐microglobulin (Fig. 6H). Hepatocytes within hTELi were functionally active as shown with the amount of serum human albumin secretion and the capacity of mice with hTELi to exhibit the human‐specific capacity to metabolize debrisoquine further confirms the presence of a significant number of surviving functional human hepatocytes in hTELi. Human albumin secretion increased with more implants: mice that hosted six hTELi implants had higher serum human albumin levels (Fig. 6J). We therefore implanted six constructs per mouse to determine the xenobiotic metabolizing capacity of hTELi and injected debrisoquine, which is known to be differentially metabolized by human hepatocytes into mice that had been implanted with LOU 4 weeks prior 24. After mass spectrometric analysis 24 at the 4‐hour time point, a significant increase in the human metabolite 4‐OH‐debrisoquine was detected (Fig. 6K; supplemental online Fig. 6).

### TELi Contained Important Components of the Liver at 13 Weeks and Contributed to Hepatic Function in Mice With a Lethal Form of Arginase Deficiency

Actin^GFP^ 16 LOU were implanted 6 weeks before induction of liver failure through tamoxifen induction in the Fah−/− Fah−/−Rag2−/−Il2rg−/−Arg^flox/flox^ UBC‐Cre/ERT2 17. This mouse model was previously reported to be uniformly lethal by 21 days after induction in female mice. Female mice that received TELi implants 6 weeks before induction of arginase deficiency survived a significantly longer period than reported historical control: TELi were harvested from healthy appearing mice with a normal ammonia level at day 35 after induction. Histologic analysis demonstrated that at 13 weeks (6 weeks after LOU implantation and a further 5 weeks after induction of arginase deficiency with tamoxifen administration), as at the 40 week time point described previously, TELi contains hepatocytes and bile duct structures (Fig. 7A, 7B). Ki67 staining identified sparse positive cells at 13 weeks ( supplemental online Fig. 7). There were significantly reduced but identifiable levels of urea in the native livers of the induced mice that hosted six TELi constructs each (Fig. 7D). We therefore do not claim that the hepatic function that resulted in normalized ammonia levels in these mice resulted entirely from the implanted TELi, and from this experiment it is not possible to distinguish the degree to which each hepatocyte population contributed. We chose this model because many animal models of the inborn errors of metabolism are not conditional, and it would be challenging to attempt the implantation of TELi in models, which are usually fatal in the first postnatal weeks; however, although this was the best available model, it did not result in total abrogation of native liver function in our hands. The serum amino acids at harvest, unlike those reported for this model without any implanted TELi for salvage 23 showed lower levels of ornithine (34.58 ± 23.7 µmol/L) but not arginine or citrulline, which were close to the control values in the urea cycle‐related amino acids ( supplemental online Table 2). Branched chain amino acids isoleucine, leucine, and valine were all closer to the reported experimental values at euthanasia in induced arginase deficiency liver failure, although only isoleucine had been statistically significant in this model. Given the very low levels of urea in the livers of four of the mice, it is likely in these and possible all of the experimental induced arginase deficient mice that TELi did contribute some function from the hepatocytes contained therein, particularly in view of the evidence of secretion of albumin from both mouse TELi and hTELi and the human metabolite production from debrisoquine by hTELi. However, a more complete inducible model of liver failure will be required to make an absolute statement.

## Conclusion

Tissue‐engineered liver was successfully generated from both mouse and human tissues and contained all of the key hepatic cell types required for effective liver function. Some of these critical hepatic functions were demonstrated in TELi, including the ability of the tissue to survive and proliferate in the toxic milieu of liver failure from arginase deficiency. Although TELi appears disorganized in comparison with native liver tissue, our model avoids two difficulties of liver cell transplantation: inability to surveil the transplanted cells and cell loss in preparation of the donor cell population. In future work, longer‐term studies of TELi will determine whether this approach might result in a durable future therapy.

## Author Contributions

N.M., A.T., R.S.: conception and design, collection and/or assembly of data, data analysis and interpretation, manuscript writing, final approval of manuscript; Y.X., X.F., and G.S.L.: collection and/or assembly of data, data analysis and interpretation, final approval of manuscript; X.H., D.J., B.T., and C.W.: collection and/or assembly of data, final approval of manuscript; K.S.W.: conception and design, financial support, data analysis and interpretation, manuscript writing, final approval of manuscript; T.C.G.: conception and design, financial support, collection and/or assembly of data, data analysis and interpretation, manuscript writing, final approval of manuscript.

## Disclosure of Potential Conflicts of Interest

The authors indicated no potential conflicts of interest.

## Supporting information

Supporting Information
