# Supplementary material for: Functional Human and Murine Tissue‐Engineered Liver Is Generated from Adult Stem/Progenitor Cells
Source: Stem Cells Transl Med. 2016 Aug 30;6(1):238–48. doi: 10.5966/sctm.2016-0205 (PMC5442734; doi:10.5966/sctm.2016-0205)
Supplement: Supplementary file 1 — Supporting Information [file SCT3-6-238-s001.pdf]

Supplemental Information - Grikscheit et al.

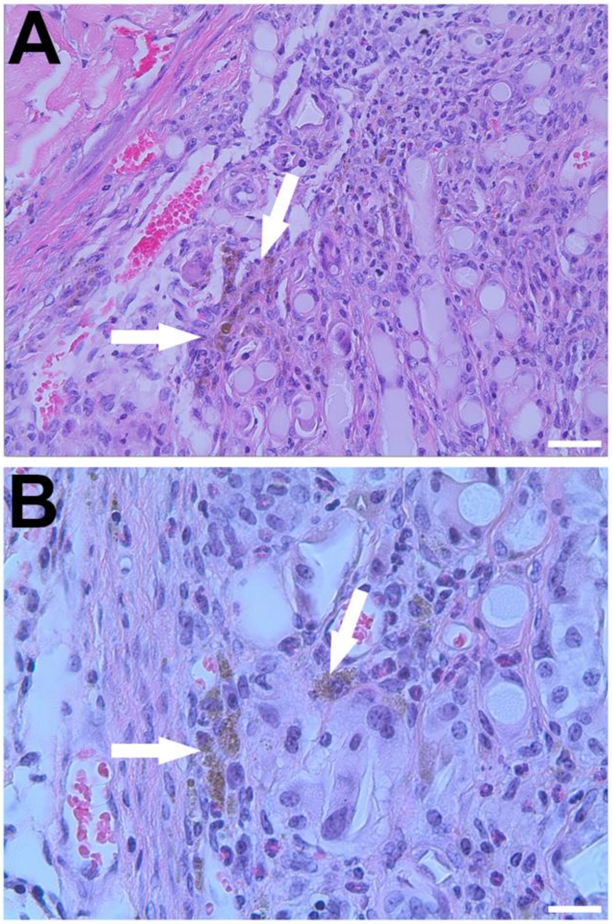

Figure S1

H&E staining demonstrates cholestasis in some hepatocytes within TELi (A-B, arrowheads). Scale bar = A, 50μm and B = 25μm.

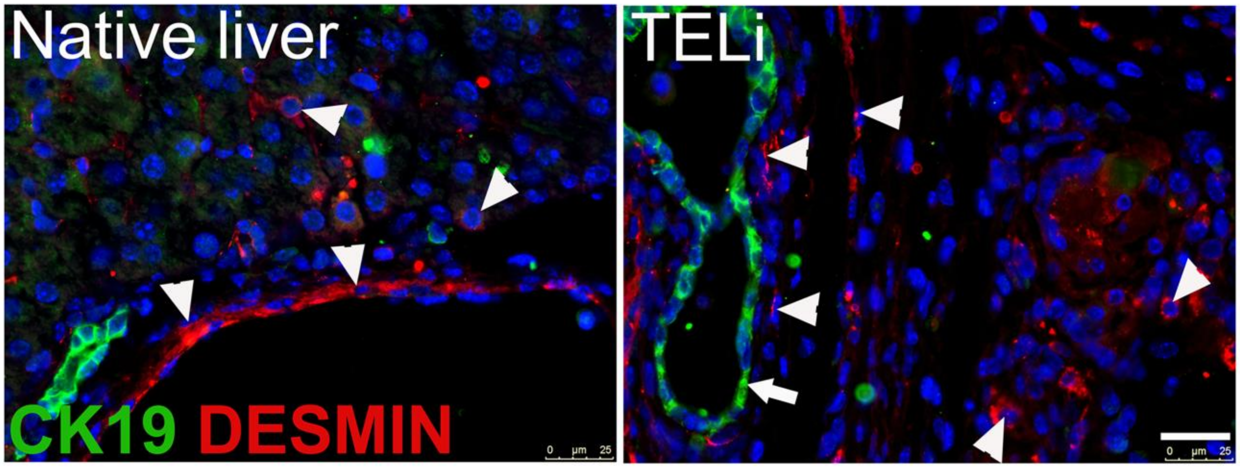

Figure S2

Immunofluorescence co-staining for Desmin (stellate cell marker) and CK19 in native liver and TELi.

Arrowhead points to positively stained cells. Nuclei stained with DAPI (Blue). Scale bar = 25μm.

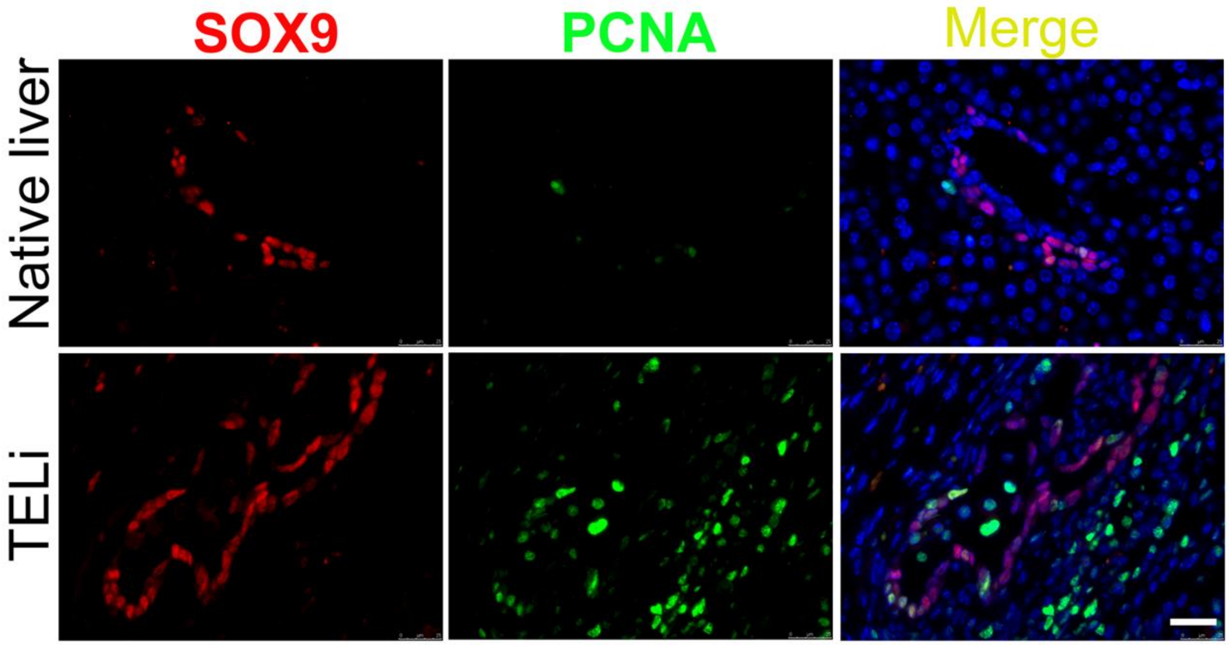

Figure S3

Biliary epithelium is marked by immunofluorescent staining for Sox9 in both TELi and native liver. Co-immunofluorescent staining with PCNA demonstrates some cells that are positive for both markers. Scale bar = 25μm.

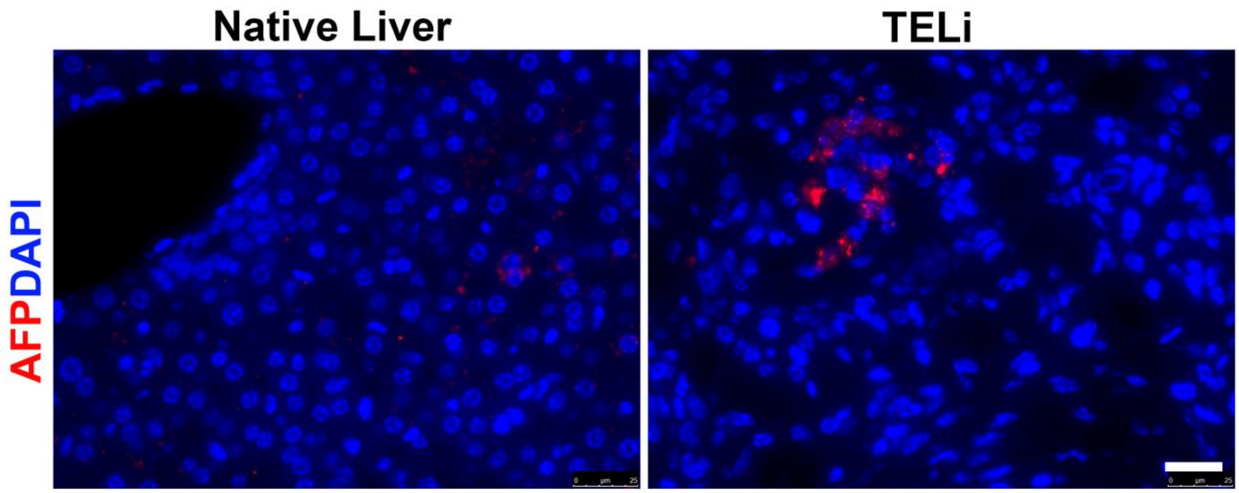

Figure S4

Immunofluorescence co-staining for AFP (Alpha- fetoprotein) in native liver and TELi. Nuclei stained with DAPI (Blue). Scale bar = 25μm.

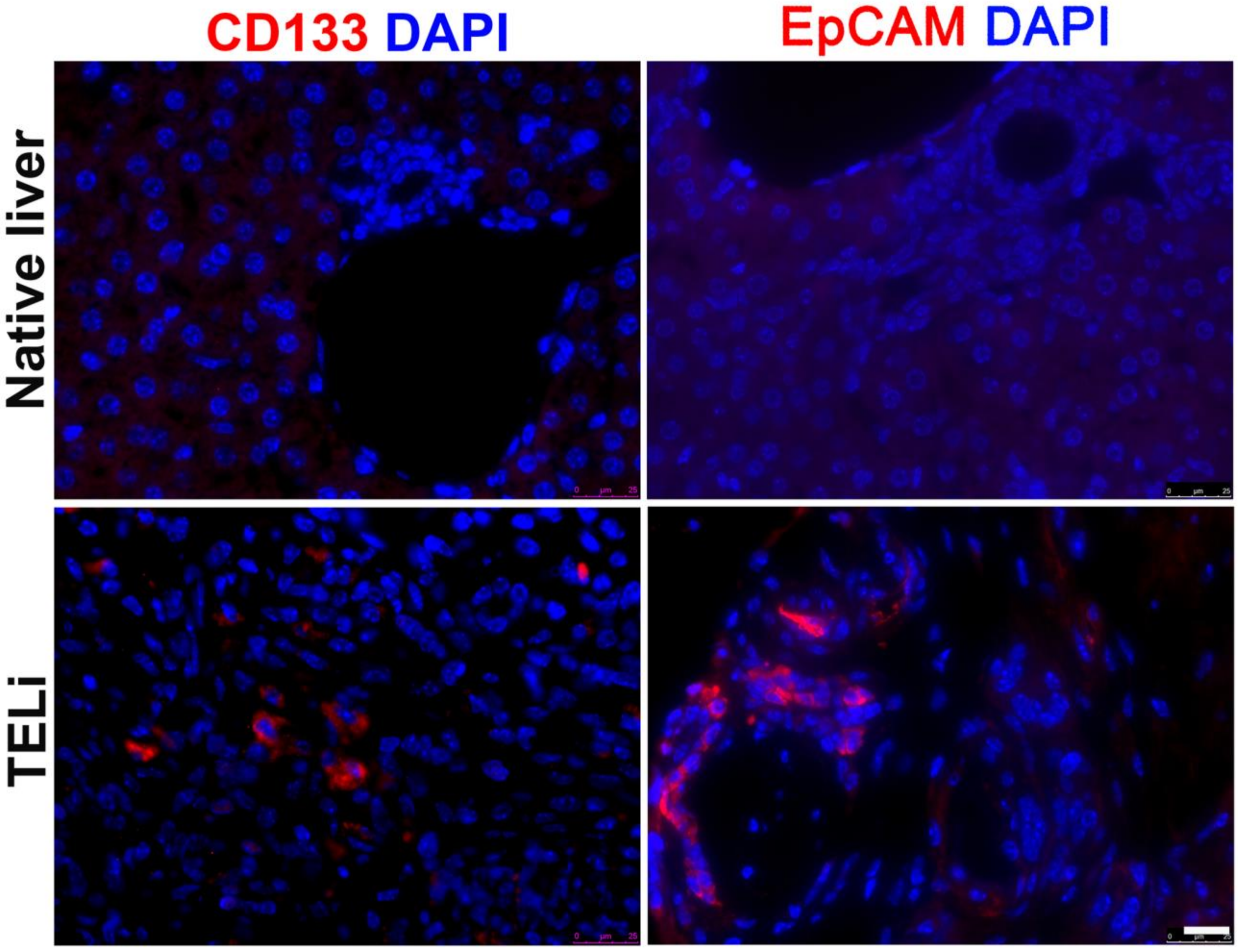

Figure S5

Immunofluorescence co-staining for CD133 and EpCAM (progenitor cell markers) in native liver and TELi.

Nuclei stained with DAPI (Blue). Scale bar = 25μm.

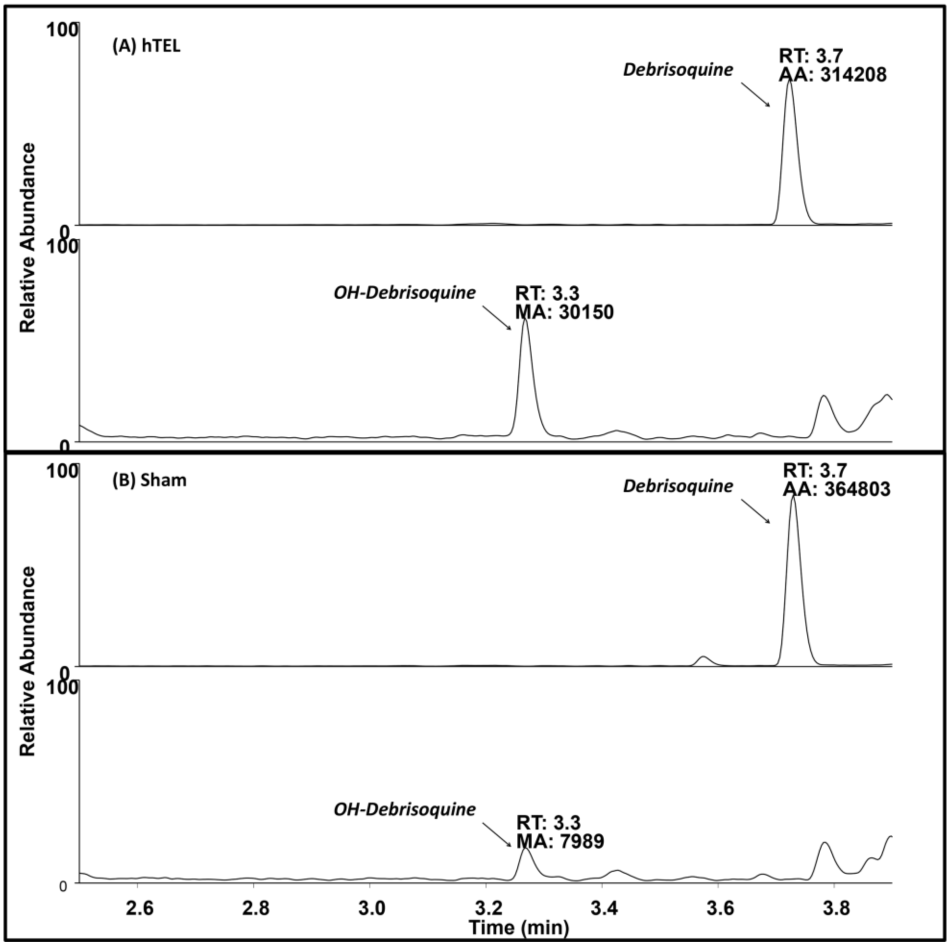

Figure S6

(A) Mass spectrometric analysis of serum from mouse that had six implanted hTELi for 4 weeks. (B) Analysis of control mouse serum that had no implanted hTELi.

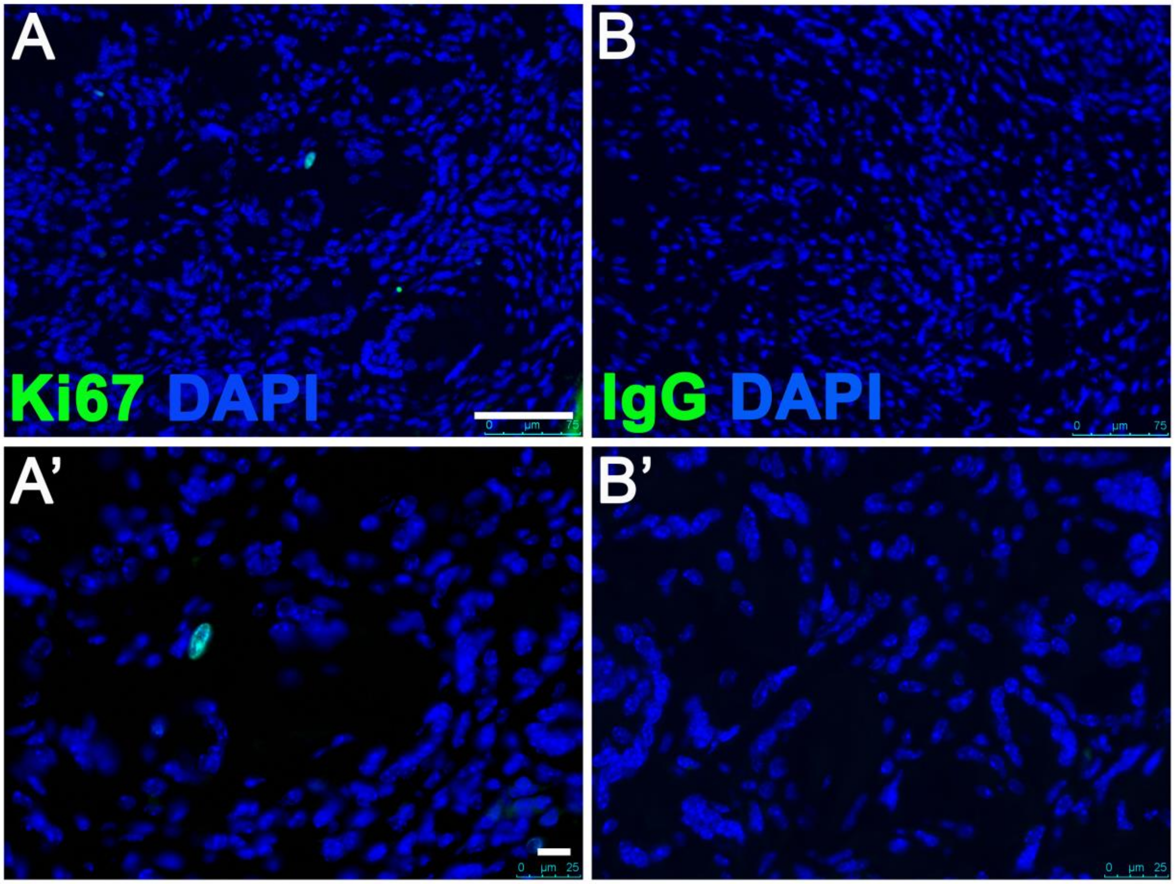

Figure S7

Immunofluorescence staining for Ki67 in 13-week TELi A, A', Scale bars 75μm and 25μm compared to IgG controls (B, B')

Supplemental Tables
Table S1

Primer sequences		
**Gene**	**Forward**	**Reverse**
Albumin	5′catgccaaattagtgcagga3′	5′gctggggttgtcatctttgt3′
Beta actin	5′tgacaggatgcagaaggaga3′	5′cgctcaggaggagcaatg3′

Primary Antibody List			
**Antibody**	**Manufacturer**	**Species**	**Dilution**
Albumin	Nordic	rabbit	1:100
Cytokeratin 19	Gift from Dr. Friedman, UPEN	rabbit	1:200
Cytokeratin 8	DSHB, University of Iowa	rat	1:100
HNF4 alpha	Perseus proteomics	mouse	1:100
αSMA-Cy3	Sigma-Aldrich	mouse	1:100
CD31	BD Pharmingen	mouse	1:100
PCNA	Vector lab	mouse	1:50
Ki67	Thermo Scientific	rabbit	1:100
Beta-2 microglobulin	Abcam	rabbit	1:50
GFP	Abcam	chicken	1:100
**Secondary Antibodies**			
Anti-Rat Cy3	Jackson Immuno Research lab	Goat	1:200
Anti-Mouse Cy3	Jackson Immuno Research lab	Goat	1:200
Ant-Rabbit Cy3	Jackson Immuno Research lab	Goat	1:200
Ant-Mouse Cy5	Jackson Immuno Research lab	Goat	1:200
Anti-Rabbit Cy5	Jackson Immuno Research lab	Goat	1:200
Anti-Mouse FITC	Jackson Immuno Research lab	Goat	1:200

Table S2

Monoamino, Monocarboxylic		Branched Chain			Mercapto		Heterocyclic
Alanine	Glycine	Isoleucine	Leucine	Valine	Cysteine	Taurine	Prolene
246.5 +/- 82.1	89.5 +/- 13.4	48.0 +/- 16.0	84.2 +/- 32.9	112.8 +/- 35.2	3.4 +/- 0.8	374.0 +/- 111.7	60.8 +/- 10.4

Diamino, Monocarboxylic		Monoamino, Dicarboxylic		Hydroxy		Carboxamide	
Histidine	Lysine	Glutamic acid	Aspartatic acid	Serine	Threonine	Asparagine	Glutamine
36.1 +/- 11.0	184.5+/- 56.1	39.9 +/- 20.8	2.2 +/- 1.8	74.3 +/- 25.9	70.1 +/- 20.0	27.2 +/- 6.7	516.9 +/- 170.6

Aromatic		Thioether	Urea Cycle-Related		
Phenylalanine	Tyrosine	Methionine	Arginine	Citrulline	Ornithine
38.2 +/- 9.2	221.7 +/- 40.3	34.2 +/- 7.6	125.1 +/- 65.3	58.7 +/- 15.8	34.6 +/- 23.8
